# Favorable outcome of a patient with an unclassifiable myelodysplastic syndrome/myeloproliferative neoplasm treated with allogeneic hematopoietic stem cell transplantation

**DOI:** 10.1177/2050313X20988413

**Published:** 2021-01-22

**Authors:** Anette Lodvir Hemsing, Bjørn Tore Gjertsen, Signe Spetalen, Lars Helgeland, Håkon Reikvam

**Affiliations:** 1 Section of Hematology, Department of Medicine, Haukeland University Hospital, Bergen, Norway; 2 Department of Clinical Science, University of Bergen, Bergen, Norway; 3 Department of Pathology, Oslo University Hospital, Oslo, Norway; 4 Department of Pathology, Haukeland University Hospital, Bergen, Norway; 5 Department of Clinical Medicine, University of Bergen, Bergen, Norway

**Keywords:** Mutational profile, myeloproliferative, myelodysplastic, overlap syndrome, approaches, allogeneic hematopoietic stem cell transplantation

## Abstract

The entity myelodysplastic syndrome/myeloproliferative neoplasm overlap syndrome is characterized by the coexistence of both myeloproliferative and myelodysplastic features in the bone marrow. Risk assessment and treatment recommendations have not been standardized, and clinicians rely on updated patient studies and reviews to make decisions for treatment approaches. Histopathological features have traditionally been important, although in the last decade, several studies have reported mutational profiles of this rare disease. Here, we present a case, wherein the patient presented with leukocytosis and the diagnostic work-up revealed features of myelodysplastic syndrome/myeloproliferative neoplasm overlap syndrome. Mutational profiling revealed mutations in four genes associated with myeloid malignancies, namely, *EZH2, CUX1, TET2*, and *BCOR*. After initial therapy with hydroxyurea and interferon-α, the patient underwent allogeneic hematopoietic stem cell transplantation, with reduced intensity conditioning and a matched sibling donor. He had no signs of relapsed disease 2 years after the transplant. Based on the patient outcome, we summarize the diagnostic and therapeutic approaches for patients diagnosed with myelodysplastic syndrome/myeloproliferative neoplasm overlap syndrome, and review the current literature, emphasizing the role of genetic mutations and allogeneic hematopoietic stem cell transplantation. Larger and more detailed clinical studies are strongly needed to optimize and standardize diagnostic and therapeutic approaches for this disease.

## Introduction

Myelodysplastic syndrome (MDS)/myeloproliferative neoplasm (MPN) overlap syndrome is a rare disease, recognized by the 2016 World Health Organization (WHO) classification of myeloid malignancies.^1^ It includes chronic myelomonocytic leukemia (CMML), atypical chronic myeloid leukemia (aCML), juvenile myelomonocytic leukemia (JMML), MDS/MPN with ring sideroblasts and thrombocytosis (MDS/MPN-RS-T), and MDS/MPN-unclassifiable (MDS/MPN-U). By definition, these cases show morphologic myeloproliferative features and signs of dysplastic bone marrow changes. The karyotype is often normal or shows abnormalities similar to MDS. Cases of CMML display peripheral blood monocytosis and dysplasia in one or more myeloid lineages. aCML is characterized by dysgranulopoiesis along with peripheral blood leukocytosis, mimicking chronic myeloid leukemia (CML). These patients are by definition *BCR–ABL1* negative, and the classical MPN mutations—*JAK2, CALR*, and *MPL*—are usually absent.^1,2,3^ The diagnostic work-up of MDS/MPN overlap syndrome is challenging due to heterogeneity and similarity to other MDS and MPN entities, for example, chronic neutrophil leukemia (CNL), accelerated phase of MPN, or prefibrotic primary myelofibrosis (prePMF).^4^ Thus, the use of myeloid mutation panels should be encouraged for proper classification of prognoses and identify subgroups that would benefit from targeted treatment.^3,5^

No standard treatment or protocol exists for the patients with MDS/MPN overlap syndrome. Allogeneic hematopoietic stem cell transplantation (allo-HSCT) has been suggested as the only curative option for eligible patients with a suitable donor.^3,6^ Here, we present the case of a 62-year-old man, who was diagnosed with MDS/MPN-U, and harbored mutations in four genes associated with myeloid malignancies: *EZH2, CUX1, TET2*, and *BCOR*. He was initially treated with induction therapy and then successfully allografted with a matched sibling donor.

## Case presentation

The patient was a 62-year-old man who was previously a smoker and had a medical history of hypertension. During a routine health check, a complete blood count was performed, revealing a severe leukocytosis (white blood cell (WBC): 62 × 10^9^/L). During hospitalization, he did not show any discomfort or signs of illness. The only constitutional symptom was light sweating during night over the previous 3 months, which had possibly worsened over the last week. His vital signs were normal. Clinical examination revealed a palpable spleen and three fingerbreadths below the costal margin. Chest radiography was normal. The spleen was 17.3 cm in length by ultrasonographic measurement, and no hepatomegaly was noted. The results of the blood tests at time of hospitalization are demonstrated in Table 1.

**Table 1.** Laboratory findings at diagnosis.

Parameter	Value	Reference value
Hemoglobin	12.3 g/dL	13.4–17.0 g/dL
WBC	64.1 × 10^9^/L	3.5–11.0 × 10^9^/L
Neutrophils including band forms	57.8 × 10^9^/L (90%)*	1.7–8.2 × 10^9^/L
Monocytes	5.9 × 10^9^/L (9.2%)*	0.04–1.3 × 10^9^/L
Platelets	441 × 10^9^/L	145–348 × 10^9^/L
Reticulocytes	0.086 × 10^12^/L	0.030–0.100 × 10^12^/L
MCV	99 fL	82–98 fL
Haptoglobin	1.71 g/L	0.50–2.10 g/L
Ferritin	701 μg/L	34–300 μg/L
Cobalamin	1177 pmol/L	175–700 pmol/L
Creatinine	81 μmol/L	60–105 μmol/L
LDH	744 U/L	105–205 U/L
Albumin	46 g/L	39–48 g/L
APTT	39 s	28–40 s
PT-INR	1.1	0.8–1.2
Fibrinogen	3.8 g/L	2.0–4.0 g/L
D-dimer	0.50 mg/L FEU	<0.50 mg/L FEU

WBC: white blood cell; MCV: mean corpuscular volume; LDH: lactate dehydrogenase; APTT: activated partial thromboplastin time; PT-INR: prothrombin time–international normalized ratio.

* Percentage of total white blood cell count.

The table indicates the hematological and biochemical findings at initial diagnosis.

**Figure 1.**
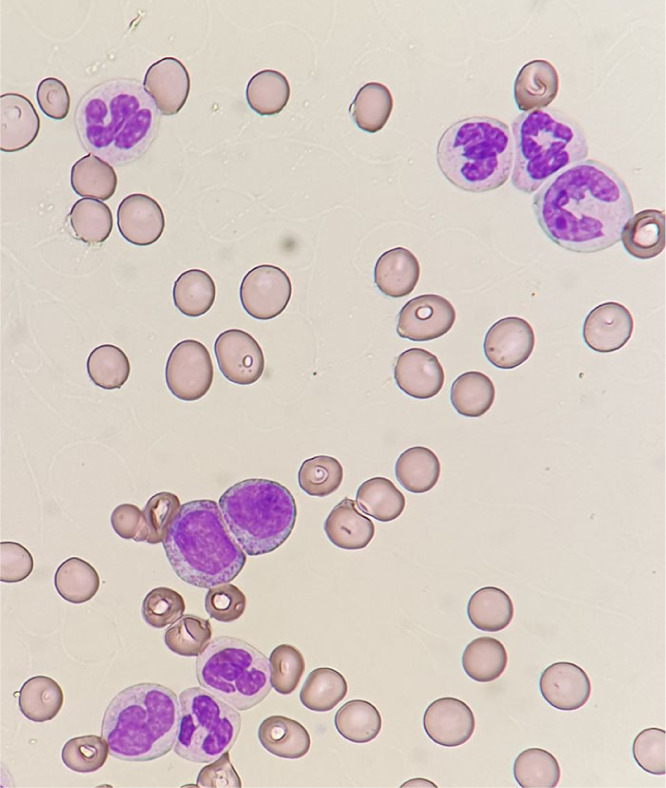
Blood smear at diagnosis. Hematoxylin and eosin–stained blood smear at diagnosis. The smear revealed neutrophils at all stages of differentiation; however, most mature neutrophils were without any clear signs of dysplasia.

The blood smear revealed neutrophils at all stages of differentiation, mostly mature neutrophils without any clear signs of dysplasia (71% neutrophils, 10% band forms, 8% metamyelocytes, and 5% myelocytes). There were 1% blasts and no basophils or monocytes (Figure 1).

**Figure 2.**
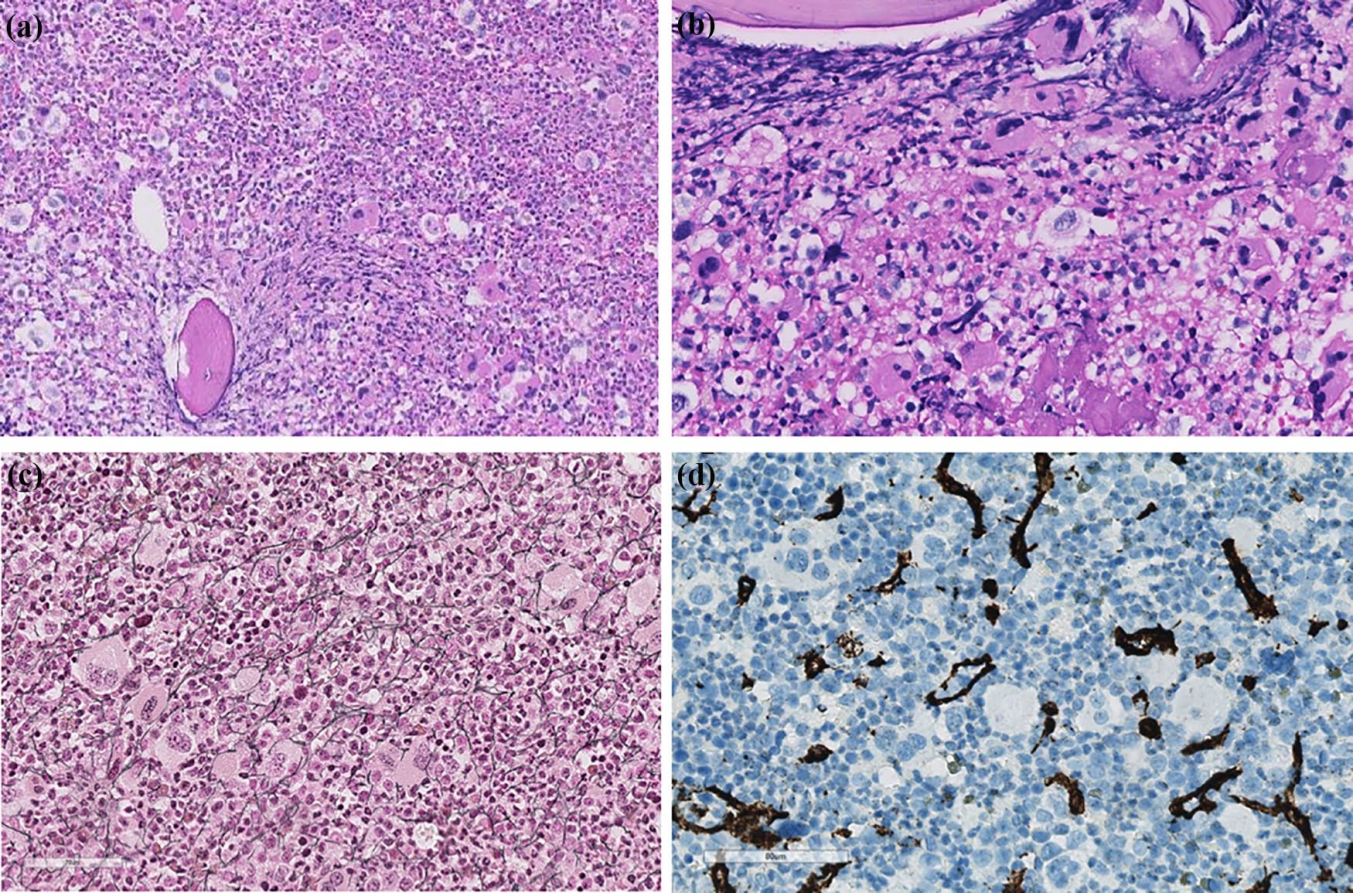
Bone marrow biopsy at diagnosis. Microphotographs of bone marrow biopsy taken at diagnosis. (a) Lower magnification view showed hypercellular bone marrow with maturing granulopoiesis, many megakaryocytes, and sparse erythropoiesis. The morphology resembled chronic myeloid leukemia. (b) Higher magnification revealed focal clustering of megakaryocytes with dysmorphic features. (c) Reticulin staining demonstrated slight reticulin fibrosis (grade 1). (d) Immunostaining for CD34 was positive in the sinusoids, but almost no positivity was observed in hematopoietic cells.

Based on the results of the blood investigations, the patient was suspected to be suffering from CML. A bone marrow examination was performed, which confirmed expanded myelopoiesis without significant dysplasia and presence of 1% blasts. As expected, mutation analyses for *JAK2, CALR*, and *MPL* were negative. Moreover, fluorescence *in situ* hybridization (FISH) analysis for the Philadelphia chromosome, t(9,22), and real-time polymerase chain reaction (RT-PCR) for *BCR–ABL1* fusion transcripts, which was initiated before the results of FISH analysis due to high clinical suspicion of CML, also turned out negative. Furthermore, G-banding demonstrated a normal male karyotype (46,XY), and FISH analysis for *PDGFRA, PDGFRB, PCM1-JAK2*, and *FGFR1* rearrangements was negative. Bone marrow biopsy confirmed signs of MDS/MPN, with dominant mature granulocytopoiesis and an increased number of individually spread atypical megakaryocytes (Figure 2).

To further confirm the diagnosis, analysis of a myeloid mutation panel (TruSight Myeloid Sequencing Panel, Illuminas, USA) was performed on the bone marrow aspirate. The panel confirmed clonal hematopoiesis in the presence of *EZH2* (variant allele frequency (VAF) 93.5%), *CUX1* (VAF 83.5%), *TET2* (VAF 53.9%), and *BCOR* (VAF 12.6%) mutations. These gene mutations are commonly observed in MDS/MPN overlap syndrome, but they are not specific for any entity within the syndrome.^5,7^ According to the 2016 WHO classification system for myeloid malignancies, we classified the patient as having MDS/MPN-U.^1^

**Figure 3.**
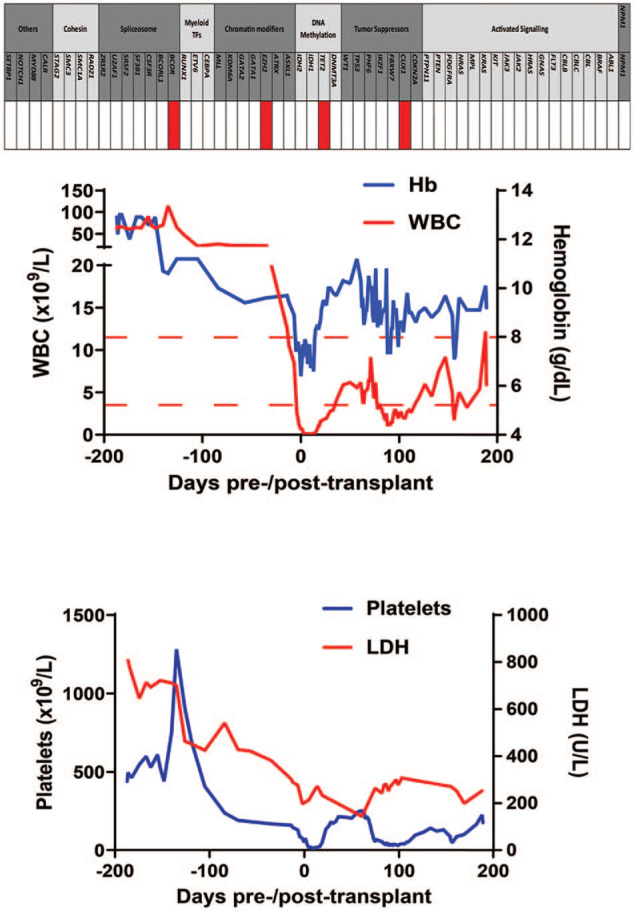
Upper panel shows the genetic mutation profile for the patient with MDS/MPN-U. The figure shows all the mutations tested in the myeloid panel and their classification. The patient harbored mutations in four genes: *TET2, EZH2, CUX1*, and *BCOR*. Lower panel shows the changes in blood parameters and lactate dehydrogenase content from diagnosis and during the treatment course. The figure demonstrates the changes in the white blood cell (WBC) count, hemoglobin (Hb), platelets, and lactate dehydrogenase (LDH) during the treatment course. Day 0 indicates the day for the allotransplant, days before day 0 are indicated by negative values, and days after day 0 are indicated by positive values.

The patient’s leukocytosis and thrombocytosis (a myeloproliferative feature of the disease) expanded over the next few weeks, and he had light constitutional symptoms. Treatment with hydroxyurea was initiated, and gradually increased to a dose of 1500 mg/day; the treatment was well tolerated. Pegylated interferon-α was added to the treatment and gradually increased to a dose of 100 mg once weekly. Consequently, while the leukocytosis improved, precursors still comprised >10% of nucleated cells. Figure 3 illustrates the blood parameters over time (Figure 3).

Reduced intensity conditioning (RIC) allo-HSCT with a matched sibling donor was performed 6 months after initial diagnosis, and the disease was stable during this period. The conditioning regimen consisted of fludarabine and treosulfan.^8^ Graft-versus-host disease (GvHD) prophylaxis was as follows: methotrexate at days +1, +3, +6, and +11, and cyclosporin A from day −1 with a target serum concentration of 200–300 µg/L.^9^ Engraftment occurred with platelets >20 × 10^9^/L on day +15, >50 × 10^9^/L on day +20, and neutrophils >0.2 × 10^9^/L on day +16. The patient developed acute skin and intestinal GvHD (grade I and IV, respectively) on day +60, which was successfully treated with steroids and addition of alpha-1-proteinase inhibitor. The therapeutic range of cyclosporin A was raised. Due to a rise in cytomegalovirus (CMV) DNA transcript levels without signs of CMV-related disease, pre-emptive intravenous ganciclovir was administered from day +90 until CMV DNA transcripts were no longer detected.^10^ Evaluation at 3, 6, 9, 12 months, and 2 years confirmed complete remission (CR) with normal blood and bone marrow smears, and donor chimerism >99%.

## Discussion

The diagnosis of *BCR–ABL1*-negative MDS/MPN mimicking CML prompts close collaboration among clinicians, pathologists, and geneticists.^3^ Our patient presented with expanded granulopoiesis without dysplasia in peripheral blood and bone marrow, and without monocytosis, eosinophilia, or basophilia. Blasts accounted for 1%–2%, and neutrophil precursors were >10% in peripheral blood. In the bone marrow, dysplasia was noted in the megakaryocytes. The lack of monocytosis excluded CMML diagnosis. Regarding bone marrow morphology, more grouped megakaryocytes and obvious fibrotic changes are expected in primary myelofibrosis, although reticulin staining demonstrated focalized increase grade 1 (Figure 2). The MPNs characteristic mutations in *JAK2, CALR*, and *MPL* were absent, although accompanying mutations were present in *TET2, EZH2, CUX1*, and *BCOR*. CNL cannot be excluded morphologically, irrespective of the findings in the megakaryocytes not being typical. Nevertheless, the number of neutrophil precursors in the peripheral blood makes CNL more unlikely and does not meet the WHO criteria. The *CSF3R* mutation, highly prevalent in CNL, was also absent.^1^

The differentiation between prePMF, aCML, and MDS/MPN-U relies on the morphological features, including the lack of peripheral cytopenia and general fibrosis in the bone marrow. The actual clinical and morphological presentation with expanded myelopoiesis is suggestive of aCML, even though there is no prominent dysplasia, except in the megakaryocytes. It has also been discussed whether dysgranulopoiesis is mandatory for the diagnosis of aCML.^3^ In a recent interesting review, Shallis and Zeidan^3^ discussed the possibility of hematopathological features in MDS/MPN overlap syndrome to be on a continuum. No statistically different karyotype findings or mutation status has been proven between aCML and MDS/MPN-U.^11^ Recently, based on the mutational landscape of 367 patients with MDS/MPN overlap syndrome, Palomo et al.^5^ found that aCML was highly associated with *ASXL1* and *SETBP1* mutations. The mutational profile of MDS/MPN-U was highly heterogeneous, with a higher frequency of *TP53* mutations.^5^ Due to the lack of dysgranulopoiesis, the diagnostic classification of MDS/MPN-U was considered most appropriate for our patient, according to the revised WHO 2016 criteria. In Table 2, we have summarized the diagnostic criteria for the most relevant myeloid malignancies considered during the diagnostic work-up for our patient.

**Table 2.** Definitions of the most relevant differential diagnosis.

	CNL	aCML	CMML	MDS/MPN-U	prePMF*
Peripheral blood	Leukocytosis ⩾25 × 10^9^/L with ⩾80% segmented neutrophils and band forms, <10% neutrophil precursors. Monocytes <1.0 × 10^9^/L. No dysgranulopoiesis	Leukocytosis due to increased numbers of neutrophils. Precursors comprising ⩾10% of leukocytes. Dysgranulopoiesis (may include abnormal chromatin clumping). Basophils <2% and monocytes <10% of leukocytes. <20% blasts.	Monocytosis ⩾1 × 10^9^/L, and ⩾10% of the total leukocytes. <20% blasts. Dysplasia in one or more myeloid lineages.	<20% blasts	Major criteria 1. Marrow findings: megakaryocytic proliferation and atypia, without reticulin fibrosis >grade 1. Increased age-adjusted cellularity, granulocytic proliferation, and often decreased erythropoiesis 2. Presence of *JAK2, CALR*, or *MPL* mutation OR presence of another clonal marker OR absence of minor reactive bone marrow reticulin fibrosis 3. Not meeting WHO criteria for *BCR–ABL1*^+^ CML, PMF, PV, or ET
Marrow findings	Hypercellular. Granulocytosis with normal maturation. Myeloblasts <5%	Hypercellular. Granulocytic proliferation and dysplasia, with or without dysplasia in the erythroid and megakaryocytic lineages. <20% blasts.	<20% blasts	<20% blasts	
Other criteria	Not meeting WHO criteria for *BCR–ABL1*^+^ CML, PMF, PV, or ET			No *BCR–ABL1* gene rearrangement	
	No rearrangement of *PDGFRA, PDGFRB*, or *FGFR1*, or *PCM1-JAK2*				Minor criteria
	Presence of *CSF3R* mutation OR persistent neutrophilia (at least 3 months), splenomegaly and no identifiable cause of reactive neutrophilia or demonstration of clonality of myeloid cells by cytogenetic or molecular studies	No *BCR–ABL1* gene rearrangement	Presence of clonal cytogenetic or molecular genetic abnormality in myeloid cells OR persistent monocytosis (at least 3 months), and other causes of monocytosis must have been excluded	Both dysplastic and proliferative features not meeting criteria for other MDS/MPN diagnoses	1. Anemia not attributed to a comorbid condition 2. Leukocytosis ⩾11 × 10^9^/L 3. Palpable splenomegaly 4. LDH increased

CNL: chronic neutrophil leukemia; aCML: atypical chronic myeloid leukemia; CMML: chronic myelomonocytic leukemia; MDS/MPN-U: myelodysplastic syndrome/myeloproliferative neoplasm-unclassifiable; prePMF: prefibrotic primary myelofibrosis; WHO: World Health Organization; LDH: lactate dehydrogenase.

The table indicates the most relevant differential diagnosis for the case presented here and their different diagnostic criteria. The table is modified from Shallis and Zeidan (Shallis and Zeidan, 2020).

* Diagnosis of prePMF requires meeting all three major criteria and at least one minor criterion.

There are no standardized risk stratification scores for MDS/MPN overlap syndromes. The International Prognostic Scoring System–Revised (IPSS-R) developed for MDS combined with cytogenetics is often used in clinical practice and studies, but its prognostic usefulness has been uncertain.^12–14^ In contrast, Mangaonkar et al.^11^ found that both IPSS-R and Global MD Anderson (MDA) model successfully risk stratified 135 MDS/MPN-U patients at 3 years follow-up. Palomo et al.^5^ found that the presence of cytogenetic abnormalities was associated with an inferior overall survival (OS) in MDS/MPN neoplasms, except for aCML. The presence of specific somatic mutations is suggested to negatively impact survival. An example is the presence of a *TET2* mutation in aCML,^15^ or *ASXL1* mutation in CMML.^16^ Palomo et al.^5^ argued that *ASXL1* mutation is not associated with poorer outcome in aCML since the disease already has an aggressive course and harbors a high percentage of *ASXL1* mutations. In their study, *EZH2* was associated with a poorer OS in patients with aCML.^5^ They also aimed to characterize a molecular subtype of MDS/MPN-U as “aCML-like,” which actually has poorer OS compared to the “CMML-like” subtype of MDS/MPN-U.^5^ Mangaonkar et al.^11^ demonstrated the adverse prognostic impact of *CBL* and *TP53* mutations in MDS/MPN-U patients. Bose et al.^12^ reported that results positive for ⩾1 gene mutation in their panel associated with worse OS, but none of them predicted an inferior outcome individually. For aCML and MDS/MPN-U diagnoses, age, severe leukocytosis, and the presence of somatic mutations are outlined as adverse factors.^2,15^

For our patient, four genes associated with myeloid malignancies were found mutated: *TET2, EZH2, CUX1*, and *BCOR*. Interestingly, they belong to four different functional classes (Figure 3), indicating cooperation and synergistic mechanisms for transformation to the malignant phenotype. In mouse models, the coexistence of inactivating mutations of *EZH2* and *TET2* accelerates the development of MDS/MPN syndromes.^17^ These epigenetic gene mutations, especially *TET2* mutations, are speculated to be the early events in adult MDS/MPN.^5,7^ The *CUX1* gene mutation on chromosome 7 is infrequently described and is not always analyzed in the mutation panels used in different case series. *CUX1* dysfunction has been proven to decrease DNA repair efficiency and is likely to cause the accumulation of somatic mutations, thereby subsequently leading to poorer survival. However, so far, it is not specially linked to a given myeloid neoplasm.^18^ The consequences of inactivating mutations of the transcription regulator *BCOR* are not clearly determined, but may result in ineffective hematopoiesis and dysplastic differentiation, especially with concurrent loss of *TET2*.^7,17^ In Table 3, we have summarized the current knowledge regarding their prevalence and potential prognostic value in different myeloid malignancies.^5,7,12,16,19–23^ However, due to the rarity of these disease entities and the low prevalence of some of the mutations, their prognostic values are uncertain. Future studies will enhance our knowledge in this regard.

**Table 3.** The prevalence and prognostic value of *TET2, EZH2, CUX1*, and *BCOR* mutations in different myeloid malignancies.

Mutation	*TET2*			*EZH2*			*CUX1*			*BCOR*		
Diagnoses	Prevalence	Prognosis	References	Prevalence	Prognosis	References	Prevalence	Prognosis	References	Prevalence	Prognosis	References
aCML	20%–35%		Palomo et al.,^5^ Deininger et al.,^7^ Savona et al.^19^	10%–30%	Inferior	Palomo et al.,^5^ Deininger et al.,^7^ Savona et al.^19^	~10%		Palomo et al.^5^	No data		
CNL	20%–30%		Deininger et al.^7^	5%–10%		Deininger et al.^7^	No data			No data		
CMML	40%–70%		Palomo et al.,^5^ Deininger et al.,^7^ Elena et al.,^16^ Savona et al.^19^	5%–15%	Inferior	Palomo et al.,^5^ Deininger et al.,^7^ Elena et al.,^16^ Savona et al.^19^	5%–10%		Palomo et al.,^5^ Deininger et al.,^7^ Elena et al.^16^	5%–10%		Deininger et al.,^7^ Tara et al.^17^
MDS/MPN-U	20%–35%		Palomo et al.,^5^ Deininger et al.,^7^ Bose et al.,^12^ Savona et al.^19^	5%–25%		Palomo et al.,^5^ Deininger et al.,^7^ Bose et al.,^12^ Savona et al.^19^	5%–10%		Palomo et al.^5^	No data		
MPN	10%–15%		Grinfeld et al.^20^	~2%		Grinfeld et al.^20^	<1%		Grinfeld et al.^20^	<1%		Grinfeld et al.^20^
MDS	~30%		Sperling et al.^21^	~5%		Sperling et al.^21^	No data			~5%	Inferior	Tara et al.^17^
s-AML	~20%		Lindsley et al.^22^	~10%		Lindsley et al.^22^	No data			~8%		Lindsley et al.^22^
De novo AML	~10%		Lindsley et al.,^22^ Papaemmanuil et al.^23^	~2%		Lindsley et al.,^22^ Papaemmanuil et al.^23^	<1%		Papaemmanuil et al.^23^	~2%		Lindsley et al.,^22^ Papaemmanuil et al.^23^

aCML: atypical chronic myeloid leukemia; CNL: chronic neutrophil leukemia; CMML: chronic myelomonocytic leukemia; MDS/MPN-U: myelodysplastic syndrome/myeloproliferative neoplasm-unclassifiable; MDS: myelodysplastic syndrome; MPN: myeloproliferative neoplasm; AML: acute myeloid leukemia.

The table demonstrates the estimated prevalence and potential prognostic value of the different mutations found in the present patient.

The natural history of aCML suggests a median survival of 25 months, and a transformation to acute myeloid leukemia (AML) in 40% within 18 months of diagnosis.^2^ Mangaonkar et al.^11^ reported a median OS of 26 months and median AML free survival (FS) of 24 months in an unselected patient cohort with MDS/MPN-U. The only curative option proposed is allo-HSCT, although a few evidence-based recommendations have been made.^3,19^ A retrospective analysis by Onida et al.^6^ described CR in 87% of patients with aCML reported to the European Society for Blood and Marrow Transplantation (EBMT) registry between 1997 and 2006, following allo-HSCT for aCML, 5 years OS in 51% patients and a relapse FS in 36% patients. Kurosawa et al.^14^ reported a 3-year OS for 48.3% patients in a cohort of 86 patients with MDS/MPN-U treated with allo-HSCT between 2001 and 2017 in Japan using registry data. The OS was worse in patients >50 years of age and those with disease progression according to the International Working Group (IWG) response criteria for MDS at the time of allo-HSCT. Both relapse and non-relapse mortality rates were approximately 25%. Mutational profiles of patients were not assessed.^14^

Despite being asymptomatic and in low-risk categories according to both the IPSS-R and the MDA model, our patient’s age, low hematopoietic cell transplantation–specific comorbidity index (HCT-CI),^24^ the adverse prognostic factors related to the mutation profile, and the availability of a suitable donor led to a relatively rapid allo-HSCT. Allo-HSCT was accomplished during the chronic phase of the disease, which is regarded favorable.^4,14^ In recent reviews, both Smith et al.^13^ and Shallis and Zeidan^3^ emphasized risk stratification and incorporation of genetic data for prognostic models in MDS/MPN overlap syndrome. It must be emphasized that the decision to initiate treatment and to transplant in the setting of a low-risk and stable-phase disease is not straightforward and should be the result of a thorough overall assessment.

Limited data exist on treatment up-front allo-HSCT. For our patient, the main goal was to avoid proliferative symptoms and progression to the blast phase. The choices of administering interferon-α and hydroxyurea were empirical, and dosages were guided with near normalization of leukocyte counts. Interferon-α is rarely mentioned as a therapeutic option in case series,^11,14^ although it has demonstrated efficacy in classical MPNs to reduce WBC with tolerated side effects.^25^ There were no *JAK2* or *CSF3R* mutations to support the use of ruxolitinib.^2^ There are ongoing studies regarding selective inhibitors of *EZH2* in myeloid malignancies,^26^ although this approach was not available in this setting. There was no increase in blasts in the marrow or dominant cytopenia, which would have prompted the use of a hypomethylating agent as a bridge to allo-HSCT.^2^ Due to the age of the patient, an RIC regimen was chosen.^8^

The MDS/MPN International Working Group (MDS/MPN IWG) published proposed response criteria for MDS/MPN in 2015,^19^ trying to unite possible dysplastic as well as proliferative aspects of the heterogeneous group. The 3-month evaluation after RIC allo-HSCT showed complete remission according to these defined response criteria. However, there is no possibility of monitoring minimal residual disease (MRD) for MDS/MPN patients although *TET2, EZH2*, and *CSF3R* are all potential markers.^4^

## Conclusion

This case report highlights the challenging diagnostic work-up required for MDS/MPN overlap syndrome. Close collaboration among clinicians, pathologists, and geneticists is essential. It is challenging since there are no standardized diagnostic or therapeutic options for this disease entity, and that somatic mutations would be incorporated in the risk assessment and considered while choosing a treatment strategy; however, their applicability in clinical settings may not be immediate. Mutational profiling will be a part of the MRD-assessment post-allo-HSCT in the future. Through our report, we show that RIC allo-HSCT is feasible in patients with MDS/MPN-U, and that complete remission is achievable.
